# Regenerative medicine owes to microsurgery

**DOI:** 10.1186/s42826-023-00158-3

**Published:** 2023-04-13

**Authors:** Kamran Shirbache, Hossein Nematian, Mohammad Hossein Nabian

**Affiliations:** 1 Center for Orthopedic Trans-Disciplinary Applied Research, Tehran University of Medical Sciences, Tehran, Iran; 2 Iranian Tissue Bank and Research Center, Imam Khomeini Medical Complex Hospital, Tehran University of Medical Sciences, Tehran, Iran; 3 Shariati Hospital, Jalal-e-Al-e-Ahmad Hwy, Tehran, Iran

**Keywords:** Regenerative medicine, Tissue engineering, Translational animal models, Microsurgery achievement

## Abstract

New findings in regenerative medicine have always been combined with numerous animal studies. Therefore, choosing the right translational animal model plays an important role in transferring as much basic knowledge as possible to clinical application in this field. Since microsurgery has many capabilities to perform precise interventions on small animal models and facilitates other regenerative medicine procedures, based on scientific articles, we believe that the key to the flourishing of regenerative medicine in the clinic is the use of microsurgery.

## Background

Considering the two main categories of regenerative medicine: cell therapy (without scaffold) and tissue engineering (with scaffold), the phrase “regenerative surgery supported by tissue engineering” has been attributed to the second group. In our view, due to the undeniable benefits of microsurgery in the advancement of tissue engineering and regenerative medicine (TERM) and its translation to human studies, "regenerative microsurgery" is a more correct phrase [1].

## Main text

To have transferred as much theoretical knowledge into practical solutions in TERM, choosing the right translational animal model is essential. As recently approved basic science achievements in clinics including blood transfusion, liver cell regeneration, and kidney transplant, the great goal of TERM is to transfer other controversial capabilities such as Central Nervous System damage repairment, Myocardiocyte regeneration, etc. from bench to bedside [2].

Interestingly, one prominent criterion of fateful animal studies entering the ultimate phase of clinical approval is high replication. Although large animals seem to have more replication, other advantages of small animal models, such as availability, easier handling, more convenient management of the animal's environment, and shorter life span to follow up the intervention until the end, considered these creatures as a favorable model in TERM. Similar hormonal mechanisms of osteoporosis and bone repair in rats, inducing osteoporosis in them with a simple ovariectomy and creating other mechanical defects in cases like growth plate fractures, coronary arteries blockage, and liver injury, through microsurgery are some obvious breakthroughs of microsurgery for researchers [3].

According to the three principles of Replacement, Reduction, and Refinement (3Rs) in animal studies, it is preferable to use small models, especially for initial trials requiring a large number of samples and several repeats of interventions with different types. Then a small size large animal study could be done for final confirmation. Obviously, for a successful intervention in the small field of surgery on these animals, microsurgery apply is a must [3, 4].

In addition to the translational animal model reasons, microsurgery benefits include other aspects. Stem cell culture and the final product management needs microscopic techniques for insertion, the same as xenogenic cell cultures producing growth factors for human grafts. Also, growth factors delivery to transplanted tissue is successful the most via biosynthesis carriers like collagen with correct kinetics, all of which require microsurgery [1].

Vascularization and neovascularization issues are the other critical points for final success in the correct embedding of the scaffold and growth factors provision. The precise placement of the scaffold in the intervention site is also essential. Noticeably, scaffolds include microscopic pores and nanofiber sheets for cell seeding and perfect nutrition which are extremely thin and sensitive to the tension of ordinary sutures; but in microsurgery, fixation is done with microscopic sutures or resorbable fibers exactly in the desired place. Additionally, the use of scaffolds in blood vessels requires the strengthening of tubular tissue against stricture by fiber, mesh, or stent all of which are conducted in small lumens with microsurgery. Nerve and small vessel grafting during organ transplantation is another microsurgery application for the transplantation of simulated organs by TERM in future studies [5] (Table 1).

**Table 1:** Some applications of microsurgery in regenerative medicine according to specific organs

1	Skin	Creating small defects, the same as burns or wound	Grafting the endothelialized reconstructed skin with the least tension and the best vascularization
2	Bone	Creating precise growth plate fracture, inducing osteoporosis in mice by ovariectomy for comparative studies	Inserting bone tissue engineered constructs for healing, injecting platelet-rich plasma for malunions
3	Nerve	Modeling of peripheral nerve damage	Microscopic nerve transplantation, assessing different bioartificial tubes
4	Vasculature	Scaffold insertion	Providing a suitable vascular bed
5	Liver	Performing partial two-thirds hepatectomy, Preforming biopsies	Hepatic cell and tissue transplantation

Honestly, there are several limitations in microsurgery, such as small animals being less similar to humans than large models, expensive equipment, unavailability of necessary infrastructure, and lack of skilled surgeons that make successful anastomosis a challenge; however, with the advancement of technology and advent of robotic-assisted surgery, soon we will witness the conquest of impossible peaks in regenerative medicine. Ambitions like cell-to-cell transplantation in CNS injuries for the repair of spinal cord lesions and precise Hiss bundle transplantation in the regenerative cardiac tissue to establish an efficient electric current in cardiomyocyte cells could be the next revolutions in medicine [3].

## Conclusion

All the above are benefits of microsurgery in TERM advancement and its more practical use. Having successes in related projects, the cooperation of an expert microsurgery team with the group is mandatory. Subsequently, we could have more astonishing improvements in the translation of research evidence in TERM from bench to bedside in the coming future.

## Data Availability

All data and materials are available in the mentioned references.

## Abbreviation

TERM: Tissue engineering and regenerative medicine
